# A Method of Implementing a 4 × 4 Correlation Matrix for Evaluating the Uplink Channel Properties of MIMO Over-the-Air Apparatus

**DOI:** 10.3390/s21186184

**Published:** 2021-09-15

**Authors:** Kazuhiro Honda

**Affiliations:** Graduate School of Engineering, Toyama University, 3190 Gofuku, Toyama 930-8555, Japan; hondak@eng.u-toyama.ac.jp; Tel.: +81-76-445-6759

**Keywords:** uplink channel, 4 × 4 correlation matrix, initial phase, Jakes’ model, multiple-input multiple-output (MIMO), over-the-air (OTA), bidirectional fading emulator, channel capacity

## Abstract

This paper presents a method of implementing a 4 × 4 correlation matrix for evaluating the uplink channel properties of multiple-input multiple-output (MIMO) antennas using an over-the-air measurement system. First, the implementation model used to determine the correlation coefficients between the signals received at the base station (BS) antennas via the uplink channel is described. Then, a methodology is introduced to achieve a 4 × 4 correlation matrix for a BS MIMO antenna based on Jakes’ model by setting the initial phases of the secondary wave sources in the two-dimensional channel model. The performance of the uplink channel for a four-element MIMO terminal array antenna is evaluated using a two-dimensional bidirectional fading emulator. The results show that the measured correlation coefficients between the signals received via the uplink channel at the BS antennas using the proposed method are in good agreement with the BS correlation characteristics calculated using Monte Carlo simulation and the theoretical formula, thereby confirming the effectiveness of the proposed method.

## 1. Introduction

Fifth-generation (5G) mobile communication systems, which will enable high speed, low latency, and large capacity, are becoming commercially available globally. Thus far, several new services that exploit these features, such as sports viewing [1] and autonomous driving [2], have been considered. To transmit high-capacity data, such as video data from a mobile terminal to a base station (BS), ultra-high-speed communication is required for the uplink channel. In the 3rd Generation Partnership Project (3GPP), 5G supports downlink and uplink peak rates of 20 and 10 Gbps, respectively [3]. Multiple-input multiple-output (MIMO) systems are essential for achieving ultra-high-speed communication [4,5]. Hence, evaluating the performance of MIMO terminals is necessary, not only for the downlink channels, but also for the uplink channels.

A straightforward method for evaluating a MIMO terminal is field testing in an actual scenario [6]. However, with field testing the repeatability and controllability of the measured data cannot be observed, and, moreover, is a very time-consuming and labor-intensive process. Hence, over-the-air (OTA) testing, which evaluates the performance of MIMO mobile terminals by creating a realistic propagation environment in the laboratory, is very important.

OTA measurement methods have been standardized by 3GPP and the Cellular Telecommunication and Internet Association (CTIA) [7,8]. In 3GPP, OTA test methodologies are classified into three categories:

(1) Reverberation chamber based methods,
(2) Two-stage methods,
(3) Multiple probe antenna based methods.

Many papers in which OTA measurements have been done using these three methods have been published. The evaluation target and issues focused on are highlighted in Table 1. The characteristics of the three OTA measurement methods are summarized as follows.

In a *reverberation chamber based method*, the chamber refers to a metallic cavity or cavities that can emulate an isotropic multipath environment that represents a reference environment for systems designed to work during fading [9,10]. The Rayleigh propagation environment in a chamber is well known as a good reference for mobile terminal systems in urban environments. The advantages of this method are its simple structure, small size, and low cost. However, it is difficult to control the multipath propagation characteristics, such as the cross-polarization power ratio (XPR), spatial correlation, and cluster, owing to the simplicity of the device.

In the *two-stage method*, the measurement is divided into two stages: first, measurements of the MIMO antenna patterns that contain all the necessary information to evaluate the antenna’s performance, such as radiation power, efficiency, and correlation, are made; then, the measured antenna patterns are incorporated with the chosen MIMO OTA channel models for real-time emulation [11,12]. The advantage of this method is the reduced number of instruments required, compared with the *multiple probe antenna based method* introduced next. However, this method requires nonintrusive complex radiation pattern measurements for accurate evaluation of the MIMO terminals.

In the *multiple probe antenna based method*, such as one with a fading emulator, a number of test antenna probes located in the chamber reproduce the channel characteristics in a controlled and repeatable manner to test MIMO performance [13,14,15,16,17]. The disadvantage of this method is that it requires many instruments, such as a probe antenna, a power divider, and a phase shifter. However, a fading emulator is capable of emulating realistic and accurate multipath environments [20,21,22,23,24].

In OTA testing, investigations primarily focus on evaluating the performance of mobile terminals in downlink channels [9,10,11,12,13,14,15,16,17]. For the uplink channel, OTA testing of a massive MIMO BS has been conducted; however, MIMO terminal OTA testing has not yet been reported [18,19]. In another paper, it was reported that there was a high correlation coefficient between the received signals in the uplink channel for a BS antenna with a separation of 5 *λ* at 2 GHz [25]. Hence, the performance of the uplink channel from the MIMO terminal must be evaluated considering the BS correlation conditions. Herein, the correlation coefficient between the signals received at the BS antennas is referred to as the BS correlation in the discussion that follows.

In one of my previous studies [21], in which the performance of the uplink channel was examined, a control method for BS correlation of a 2 × 2 MIMO system based on Jakes’ model [26] using a *multiple probe antenna based method* was proposed. In the 2 × 2 MIMO system, there are two BS antennas; therefore, the BS correlation can be controlled by using the virtual point source separation to represent the spatial correlation of the BS antennas. However, in a 4 × 4 MIMO system, there are six possible combinations of spatial correlation, owing to the different relative separations between the BS antennas when the four-element BS antennas are arranged in, for example, linear or circular arrays.

This paper presents a methodology to control the spatial correlation of the BS antennas in a 4 × 4 MIMO system based on Jakes’ model with communication via the uplink channel. First, the implementation model used to enable BS correlation with the BS antenna arrangement is described. Then, a 4 × 4 correlation matrix of a BS MIMO antenna is achieved by controlling the initial phases of the secondary wave sources depending on the BS correlation. Finally, the validity of the proposed initial phase setting method used to assess the 4 × 4 MIMO system was verified by evaluating the uplink channel using bidirectional MIMO-OTA apparatus.

The remainder of this paper is organized as follows: in Section 2 I present the propagation model for the uplink channel; in Section 3 the methodology of the proposed method is described; Section 4 shows the method for implementing a 4 × 4 correlation matrix; in Section 5 I discuss the experimental and analytical results; Section 6 concludes the paper.

## 2. Uplink Channel Model

In the downlink channel, radio waves emitted from BS antennas are reflected and diffracted by surrounding objects, such as buildings or trees, and they construct secondary wave sources, that is, scatterers, around the MIMO terminal. Furthermore, radio waves radiated from the secondary wave sources are combined to form a single coherent wave in the antennas at the MIMO terminal via different routes. Then, the incoming radio waves around the MIMO terminal generate a uniform distribution, and the correlation coefficient between the signals received at the MIMO terminal antennas is low, regardless of the distance between them [27].

One of the most common approaches to analyze a MIMO antenna system is to use Monte Carlo simulation, where many scatterers are arranged on a circle, known as Clarke’s model [28], to simulate a multipath fading channel in a mobile propagation environment; this results in the evaluation of the MIMO terminal performance [24]. A multipath propagation environment is simulated by the amplitude and phase of each wave radiated from the scatterers, which is an aggregation of all the information in the radio waves emitted by the BS antennas. A spatial fading emulator based on Clarke’s model is implemented using Monte Carlo simulation; thus, controlling the amplitude and phase of the scatterers is essential to create a realistic propagation environment.

However, for the uplink channel, the BS correlation is high regardless of the BS antenna separation because the angular spread of the incoming wave for the BS antennas is very narrow [29]. The BS correlation is known to be relatively high, even in the case of array spacings of several wavelengths in a small cell environment [25]. Moreover, the BS correlation is expected to be higher when the direction of the incident wave is parallel to the BS antenna array. Hence, when the performance of the uplink channel from the MIMO terminal is examined, the channel model must implement a high BS correlation that is different from that of the downlink channel assumed to be independent and identically distributed (i.i.d.).

**Figure 1:**
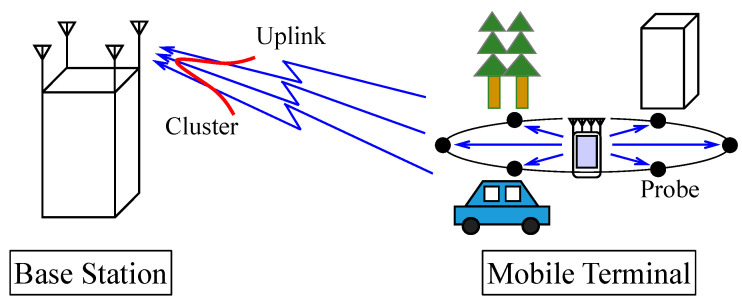
Propagation model for the uplink channel.

Figure 1 shows the propagation model for the uplink channel, wherein the radio waves emitted from the MIMO terminal are reflected and diffracted over the full azimuth by surrounding objects, such as buildings or trees, which are the relay points, that is, probes, around the MIMO terminal. Furthermore, the radio waves radiating from the probes combine to generate a single coherent wave at the BS antennas via different routes.

The BS correlation for the uplink channel generally involves the characteristics of radio wave propagation, which is represented by multipath waves and the BS antenna characteristics. In this paper, the sum of the two above-mentioned effects is attributed to the characteristics of the multipath waves, which are represented by the amplitude and phase of each wave radiating from the probes. Consequently, the area around the BS was not modeled in Figure 1.

## 3. Method to Control the BS Correlation

**Figure 2:**
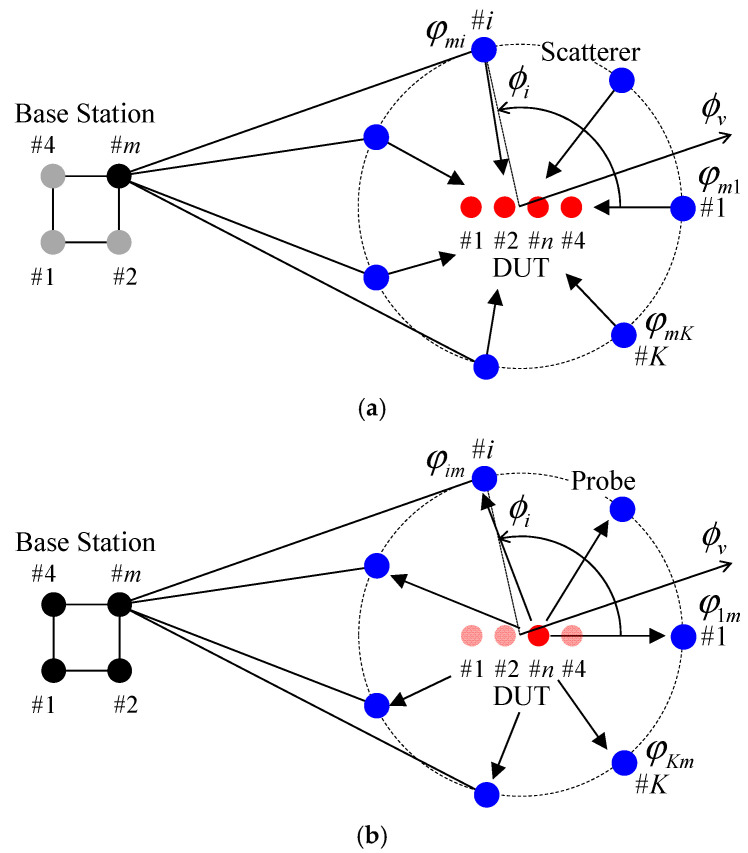
Two-dimensional channel model: (**a**) downlink; (**b**) uplink.

Figure 2 illustrates two-dimensional channel models of the downlink and uplink channels at the MIMO terminal [30]. In Figure 2, *K* secondary wave sources arranged on a circle act as the radiating antennas (scatterers) and receiving antennas (probes) for the downlink and uplink channels, respectively.

For the downlink channel, illustrated in Figure 2a, radio waves emitted from BS antennas are received by the antennas in the device under test (DUT) via the scatterers surrounding the DUT. In this paper, non-line-of-sight (NLOS) scenarios are examined; thus, Clarke’s model is used, which uniformly inputs radio waves from the full azimuth.

In the fading emulator or Monte Carlo simulation, radio waves radiated from the scatterers that surround the DUT antennas, which are controlled phases of the signal used to emulate the Rayleigh fading channel, are summed around the DUT antennas, and the desired radio environment is generated. Furthermore, when the amplitudes of the signals radiated from the scatterers are controlled according to the power spectrum of the incident wave, a cluster propagation environment can be represented.

The phase-shift of the carrier wave from the *i*-th scatterer according to the *m*-th BS antenna, $P_{mi}\left(t\right)$, is calculated as follows:

(1) $$P_{mi}\left(t\right)=2\pi f_{D}t\cos\left(\phi_{i}-\phi_{v}\right)+\varphi_{mi},$$

where $\phi_{i}$ is the azimuth angle of the *i*-th scatterer, and $\phi_{v}$ is the direction of movement of the DUT antenna. $f_{D}$ is the maximum Doppler frequency in Hertz, which is given by $f_{D}=v/\lambda$, where v is the speed of the DUT antennas and λ is the wavelength of the carrier wave. $\varphi_{mi}$, which is set by a random number, is the initial phase of the *i*-th scatterer with respect to the *m*-th BS antenna, and this can control the correlation coefficient between the channels [7].

The correlation coefficient between the signals received via the downlink by the DUT antennas is determined by the radiation pattern around the antennas. In the case of the Jakes’ model, the spatial correlation between the antennas can be expressed as

(2) $$\rho =J_{0}\left(\frac{2\pi d}{\lambda }\right),$$

where $J_{0}\left(\right)$ is a zeroth-order Bessel function of the first kind, and *d* is the distance between the isotropic antennas. As indicated in Equation (2), the correlation coefficient between the signals received at the DUT antennas depends on the geometrical phase between the antennas regardless of the initial phase of the scatterers which can be represented by another channel from the BS antenna.

Similarly, in the fading emulator, the correlation coefficient between the received signals, considering the phase difference generated by the geometrical relationship between the DUT antennas and each scatterer, is equivalent to the spatial correlation calculated by Equation (2). Hence, the geometrical relationships between the DUT antennas and the scatterers change with the position of the DUT antennas at the fading emulator, and this is very important for controlling the correlation coefficient.

In the uplink illustrated in Figure 2b, channel reciprocity theory is used to describe that radio waves emitted from the DUT antennas received by the BS antennas via the probes surrounding the DUT. As mentioned in Section 2, in this paper, the initial phase of the probes represents the BS correlation characteristics for the whole uplink channel including the path and the BS antenna properties.

The incoming signal at the BS is calculated by combining the signals from all the probes that are superimposed on the received signal and the phase-shift values of the probes. Therefore, the combined signal varies depending on the initial phases of the probes, even though the set of received signals from the probes are the same since the terminal antenna in a fading emulator does not move, which results in the generation of an incoming signal to any BS antenna. Based on the above-mentioned principle, the setting of the initial phases of the probes is very important to embody a realistic BS correlation for the uplink channel.

**Figure 3:**
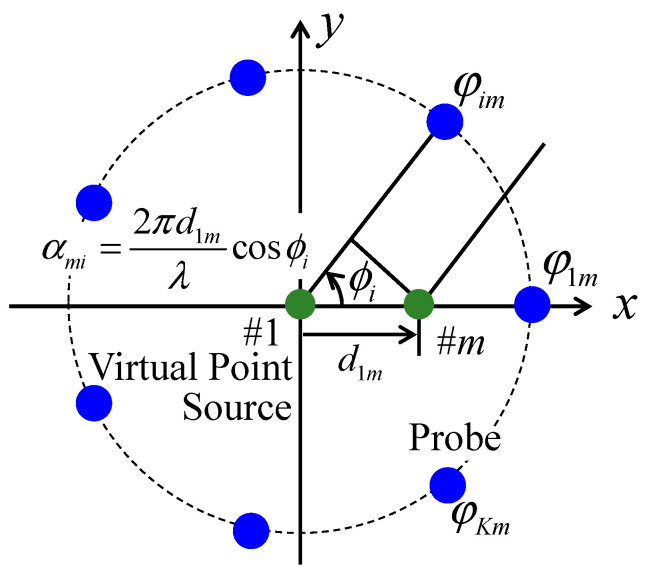
Implementation model for BS correlation.

Figure 3 shows the implementation model used to achieve the BS correlation. In Figure 3, the virtual point sources illustrated by the green circles are positioned to generate a geometrical phase difference that realizes the desired BS correlation. Therefore, it is different from the actual BS antenna arrangement and the channel model used to evaluate a MIMO antenna using the fading emulator or Monte Carlo simulation.

The received signals of each BS antenna can be observed using different initial phase matrices depending on the desired BS correlation $\Phi_{m}$. When the reference source is the virtual point source of BS #1 placed at the center of the implementation model, the virtual point source of BS #*m* is arranged at a distance *d*_1*m*_ from that of BS #1. Then, the distance *d*_1*m*_ is decided depending on the desired BS correlation using Jakes’ model. Therefore, the geometrical phase difference between the virtual point source of BS #*m* and probe #*i* $\alpha_{mi}$ is given by

(3) $$\alpha_{mi}=\frac{2\pi d_{1m}}{\lambda }\cos\phi_{i}.$$

The initial phase matrix corresponding to BS #*m* $\Phi_{m}$ is the sum of the initial phase matrix for BS #1, which is generated by a random number, and the geometrical phase difference matrix using Equation (3), and it is defined as

(4) $$\begin{array}{cc} \Phi_{m} & =\Phi_{1}+A_{m}\\ & =\left[\begin{array}{cccc} \varphi_{11}+\alpha_{m1} & \varphi_{21}+\alpha_{m2} & \cdots & \varphi_{K1}+\alpha_{mK} \end{array}\right] \end{array}$$

In Jakes’ model, spatial correlation is calculated as a function of the antenna separation. However, in this paper, it was necessary to determine the distance between the virtual point sources *d* according to the desired BS correlation. In [21], a third-order approximate formula for the relationship between the spatial correlation and antenna separation was derived via the least-squares method. However, the accuracy of the estimated formula is poor when the spatial correlation is close to 1. The spatial correlation is expected to be close to 1 in a four-element BS antenna depending on the direction of the incident wave; therefore, a high-accuracy 4 × 4 correlation matrix cannot be realized using the conventional method.

**Figure 4:**
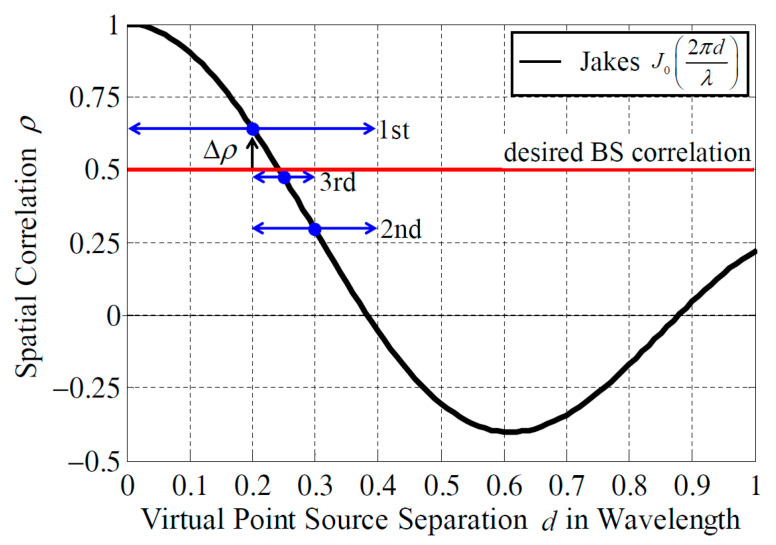
Virtual point source separation vs. desired BS correlation.

In this paper, the geometrical phase difference required to complete the desired BS correlation is determined via the bisection method using Jakes’ model, as depicted in Figure 4. First, the distance between the virtual point sources is set to the range from 0 to 0.4 *λ*, as denoted by the blue arrows in Figure 4, so that the spatial correlation satisfies 0–1. Then, the spatial correlation at *d* = 0.2 *λ*, which is the midpoint of the blue arrow, is calculated using a zeroth-order Bessel function of the first kind, as indicated in Equation (2). If the absolute value of the difference between the calculated spatial correlation and the desired BS correlation, |Δρ|, is less than the threshold value, which is a sufficiently small value, the distance between the virtual point sources is set. When it is greater than the threshold value and the calculated spatial correlation is greater than the desired BS correlation, the range of the distance between the virtual point sources is modified to the upper half of the original range. In the other case, the range is changed to the lower half. By repeating this operation, the distance between the virtual point sources that enables the desired BS correlation can be determined with high accuracy. Notably, the distance between the virtual point sources is set to 0 when the desired BS correlation is 1.

## 4. Method for Implementing a 4 × 4 Correlation Matrix

### 4.1. Corellation Characteristcs of the BS

Radio waves that arrive at the BS from the MIMO mobile terminal have a narrow angular spread and are incident from various directions depending on the position of the MIMO terminal [29]; thus, the BS correlation matrix is determined by the arrangement of the BS antennas, the direction of the incident wave, and the angular spread of the incident waves. In a 4 × 4 MIMO system, the realized 4 × 4 correlation matrix has six correlation values (*ρ*_12_, *ρ*_13_, *ρ*_14_, *ρ*_23_, *ρ*_24_, and *ρ*_34_). These correlation values are converted into the six distances between the virtual point sources, as explained in Section 3, and it is necessary to properly arrange the four virtual point sources to achieve the six BS correlations.

**Figure 5:**
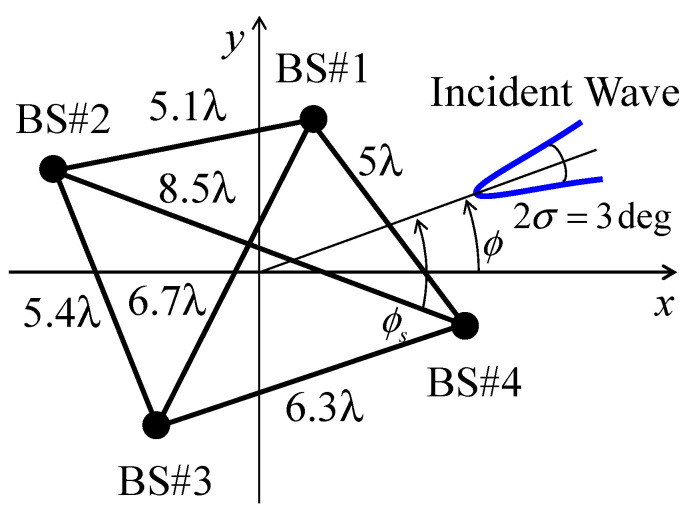
Arrangement of the BS antennas.

For generality, Figure 5 shows a model in which four BS antennas are arranged arbitrarily. In the figure, the wavelength numbers denote the distances between the antennas. The four BS antennas are isotropic antennas. When the angular spread of the incident wave is sufficiently narrow, the spatial correlation (absolute value of the complex correlation) between the antennas can be calculated as follows [31]:

(5) $$\rho =\left|\exp\left(j\frac{2\pi d}{\lambda }\cos\phi_{s}-\frac{4\pi^{2}d^{2}\sin^{2}\phi_{s}\sigma^{2}}{2\lambda^{2}}\right)\right|,$$

where *d* is the distance between the BS antennas, *ϕ_s_* is the angle of the incident wave relative to the line that connects the individual BS array, and *σ* is the angular spread of the incident wave. In this paper, the 4 × 4 correlation matrix was calculated assuming *σ* = 1.5° [29].

In the case where the incident wave angle for the *x*-axis ϕ is 0°, the spatial coefficient between BS #1 and BS #2, *ρ*_12_, is 0.987 (using Equation (5)), and the distance between the virtual point sources of BS #1 and BS #2 to realize *ρ*_12_ = 0.987 is 0.037 *λ* (using the bisection method mentioned in Section 3). Similarly, *ρ*_13_, *ρ*_14_, *ρ*_23_, *ρ*_24_, and *ρ*_34_ are 0.614, 0.805, 0.713, 0.885, and 0.947, then *d*_13_, *d*_14_, *d*_23_, *d*_24_, and *d*_34_ are 0.209 *λ*, 0.144 *λ*, 0.177 *λ*, 0.109 *λ*, and 0.074 *λ*, respectively.

### 4.2. Arrangement of Virtual Point Sources in the Implementation Model

To realize a 4 × 4 correlation matrix, the arrangement of virtual point sources that generate the initial phase in the implementation model in order to achieve the desired BS correlation is examined. It should be noted that the implementation model used to realize the BS correlation is a physical model that obtains the 4 × 4 correlation matrix using a zeroth-order Bessel function of the first kind that expresses the relationship between the spatial correlation and the antenna separation in Jakes’ model. In this paper, the two-point source model depicted in Figure 3 [21] is extended to a four-point source model.

**Figure 6:**
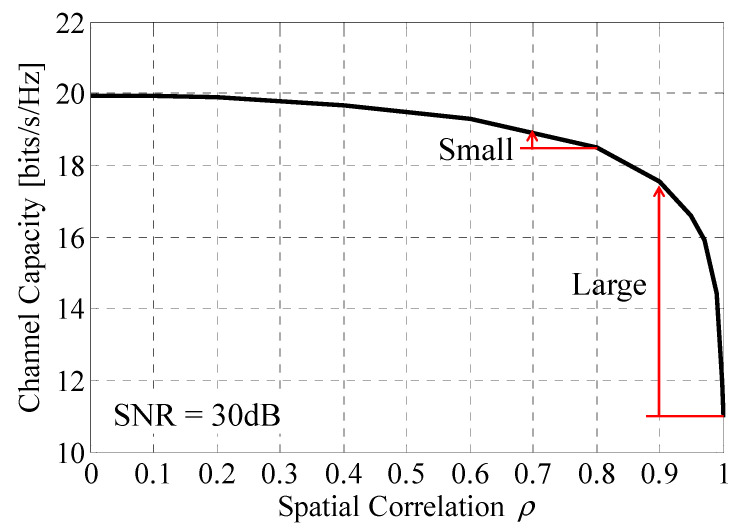
Channel capacity vs. spatial correlation.

Figure 6 shows the relationship between the channel capacity of a 2 × 2 MIMO system calculated using Shannon’s theorem and the spatial correlation [32]. The signal-to-noise ratio (SNR) is set to 30 dB. The analytical results in Figure 6 show that the impact of the change in spatial correlation on the MIMO channel capacity is very large in high-correlation situations.

In the implementation model, the distances between the six virtual point sources vary depending on the desired BS correlation. Then, there may not be a geometric arrangement of virtual point sources that represents the distances between all the virtual point sources. In this paper, to evaluate the performance of the uplink channel for MIMO terminals, the coordinates of the four virtual point sources were determined with priority given to high correlation; thus, the MIMO channel capacity using that arrangement could be close to the actual MIMO channel capacity.

**Figure 7:**
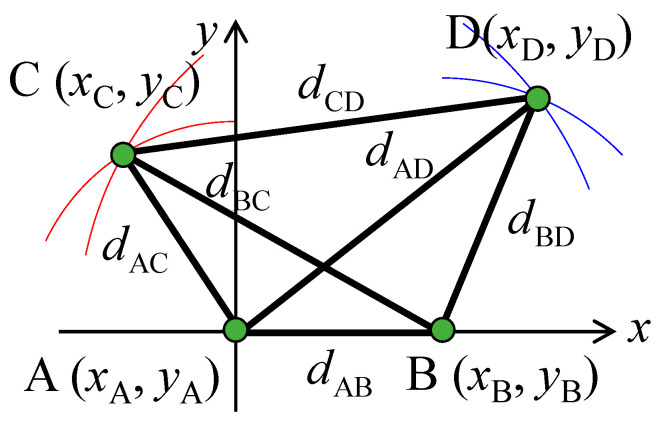
Arrangement of virtual point sources.

Figure 7 shows the arrangement of the virtual point sources in a 4 × 4 MIMO system. By placing this model in the center of Figure 3, the geometrical phase difference can be calculated using Equation (3) to generate the initial phase matrix described in Equation (4). The arrangement of the virtual point sources in Figure 7 varies in an arbitrary fashion by changing the distance relationships between the virtual point sources according to the desired 4 × 4 correlation matrix.

**Figure 8:**
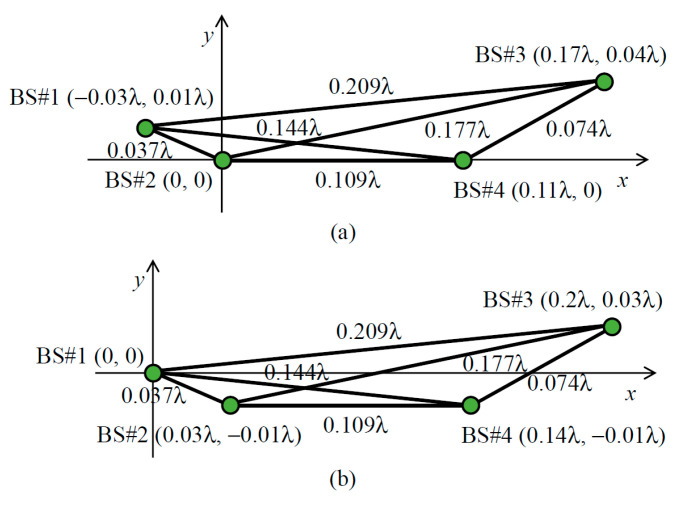
Arrangement of the virtual point sources to achieve the BS correlation at ϕ = 0° in Figure 5: (**a**) before translating; (**b**) after translating.

The relationship between the four virtual point sources (A, B, C, and D) in Figure 7 and the BS antennas (BS #1, #2, #3, and #4) is determined according to the desired 4 × 4 correlation matrix. First, the maximum distance is identified from the six distances between the four virtual point sources, and these are labeled, for example, as *d*_CD_ in Figure 7. In the case of Figure 5, *d*_13_ is *d*_CD_; thus, points C and D are either BS #1 or #3. As a result, points A and B become either BS #2 or #4, and *d*_AB_ is set to *d*_24_. Second, the maximum distance is identified from the remaining four distances, and this is set to *d*_AD_, which is the diagonal of the implementation model. In Figure 5, *d*_23_ is *d*_AD_. Therefore, points A, B, C, and D are uniquely determined to be BS #2, BS #4, BS #1, and BS #3, respectively. Finally, the coordinates of the four virtual point sources are established. The coordinates of point A (*x*_A_, *y*_A_) are used as the origin. The coordinates of point B (*x*_B_, *y*_B_) may be any point on the circumference of radius *d*_AB_ centered on point A; however, those are set to (*d*_AB_, 0) on the *x*-axis. The coordinates of point C (*x*_C_, _yC_) are the intersection of the circumference of radius *d*_AC_ centered on point A and the circumference of radius *d*_BC_ centered on point B, as illustrated by the red arcs in Figure 7. However, when *d*_BC_ > *d*_AC_ + *d*_BA_, the coordinates of point C are set to (−*d*_AC_, 0) so that *d*_AC_ and *d*_AB_ are satisfied, and *d*_BC_ is set to the longest possible value. Similarly, the coordinates of point D (*x*_D_, *y*_D_) are at the intersection of the circumference of radius *d*_AD_ centered on point A and the circumference of radius *d*_BD_ centered on point B, as illustrated by the blue arcs in Figure 7. As before, when *d*_AD_ > *d*_AB_ + *d*_BD_, the coordinates of point D are set to (*d*_AB_ + *d*_BD_, 0) so that *d*_AB_ and *d*_BD_ are satisfied, and *d*_AD_ is set to the longest possible value. The virtual point source arrangement of the implementation model in Figure 5 is shown in Figure 8a.

The maximum distance *d*_CD_ is not utilized when determining the coordinates of the four virtual point sources. This is because there may not always be a geometrical arrangement of the four virtual point sources that realizes all the distances between the virtual point sources; therefore, the shorter distances between virtual point sources, that is, a higher BS correlation, are given priority to establish the coordinates of the virtual point sources. As a result, the arrangement of the virtual point sources may satisfy all the distances required to enable the desired 4 × 4 correlation matrix. Accordingly, the arrangement of the virtual point sources, shown in Figure 8a, satisfies all the distances between the virtual point sources, resulting in the desired 4 × 4 correlation matrix.

The initial phase matrix for each BS antenna is created by calculating the geometric phase difference for the designed implementation model, as shown in Figure 8a, with reference to the initial phase matrix of BS #1. Therefore, the coordinates of the virtual point source for BS #1 is translated to the origin, as presented in Figure 8b. Using the above method, the arrangement of the virtual point sources to complete the 4 × 4 correlation matrix, which is dependent on the arrangement of the BS antennas, the direction of the incident wave, and the angular spread of the incident wave, can be determined.

### 4.3. Design of Implementation Model for the Conventional BS Antenna Arrangement

**Figure 9:**
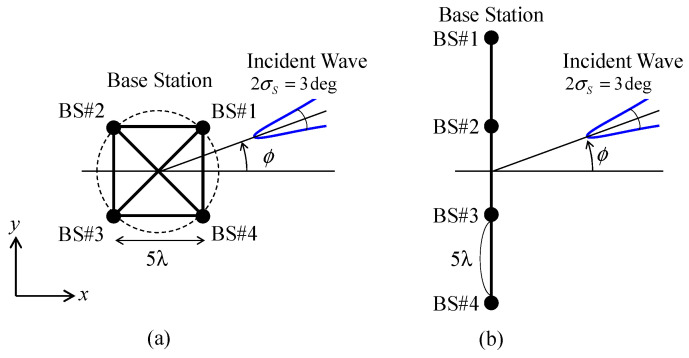
Relationship between the BS antenna arrangement and the incident wave: (**a**) circular array; (**b**) linear array.

Figure 9 shows the relationships between conventional BS antenna arrangements and the incoming wave angle ϕ. In this paper, two types of four-element BS antennas are considered: a circular and a linear array installed at equal intervals, as illustrated in Figure 9a,b, respectively [33]. The distance between adjacent antennas was set to 5 *λ*.

**Figure 10:**
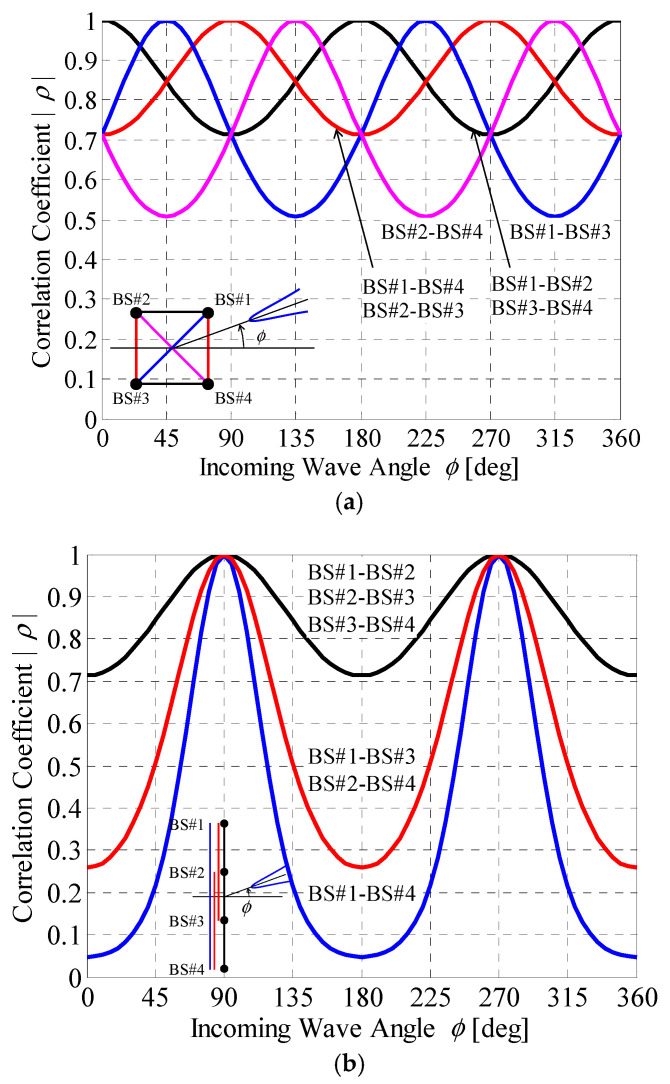
BS Correlation characteristics vs. incoming wave angle: (**a**) circular array; (**b**) linear array.

First, the 4 × 4 correlation matrix is derived using Equation (5), considering the angle of the incident wave and the distances between the individual BS antennas. Figure 10 shows the BS correlation as a function of the incoming wave angle. As shown in Figure 10, the 4 × 4 correlation matrix varies significantly depending on the BS antenna arrangement and the incoming wave angle ϕ.

In the case of the circular array, illustrated in Figure 9a, the angle of the incident wave for the BS array constructed by BS #1 and BS #2 at the incoming wave angle ϕ is the same as that constructed by BS #3 and BS #4, whereas the angle of the incident wave for the BS array constructed by BS #1 and BS #4 at ϕ is the same as that constructed by BS #2 and BS #3. Consequently, *ρ*_12_ and *ρ*_14_ coincide with *ρ*_34_ and *ρ*_23_, respectively, regardless of the incoming wave angle ϕ, and there are four types of BS correlation characteristics, as shown in Figure 10a.

However, in the case of a linear array, illustrated in Figure 9b, the angle of the incident wave for any BS array is the same; however, the distance between the BS antennas is different. The distance between BS #1 and #2 is the same as that between BS #2 and #3 or between BS #3 and #4, whereas the distance between BS #1 and #3 is the same as that between BS #2 and #4. Consequently, there are three types of BS correlation characteristics, as exhibited in Figure 10b.

**Figure 11:**
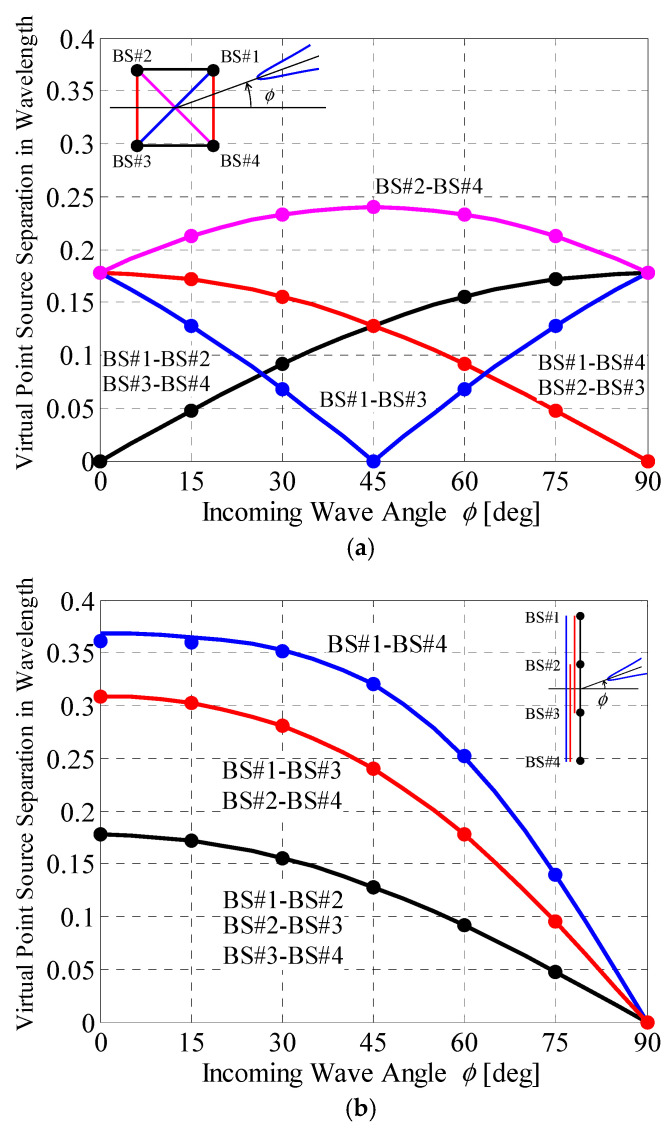
Virtual point source separation vs. incoming wave angle: (**a**) circular array; (**b**) linear array.

Second, based on the BS correlation characteristics shown in Figure 10, the distances between the virtual point sources are estimated via the bisection method using Jakes’ model. Figure 11 shows the virtual point source separation as a function of the incoming wave angle, which ranges from 0° to 90° considering the symmetry of the BS arrangement.

As described in Figure 11, the virtual point source separations according to the designed implementation model, indicated by dots in the figure, are in good agreement with the ideal distance that is required to achieve the desired BS correlation shown in Figure 10. Therefore, the 4 × 4 correlation matrix to be mounted on the MIMO-OTA apparatus to embody the desired BS correlation can be generated by the proposed method, which determines the implementation model depending on the arrangement of the BS antenna and the incoming wave angle.

**Figure 12:**
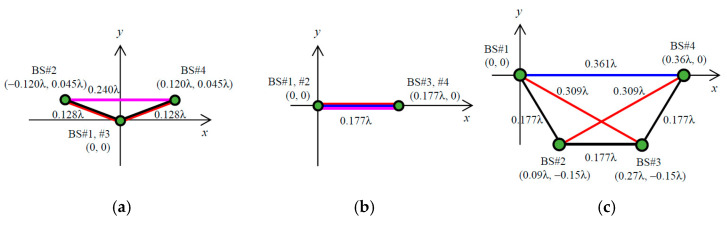
Arrangement of virtual point sources: (**a**) ϕ = 45° in a circular array; (**b**) ϕ = 0° in a circular array; (**c**) ϕ = 0° in a linear array.

As shown in the circular array, illustrated in Figure 9a, when the incoming wave angle is 45° the spatial correlation *ρ*_13_ is 1 because the array formed by BS #1 and #3 is parallel to the incident wave. Therefore, the virtual point sources for BS #1 and BS #3 have the same coordinates; therefore, there are only three virtual point sources in the implementation model. Furthermore, when the incoming wave angle is 0°, the spatial correlations *ρ*_12_ and *ρ*_34_ are 1 because two arrays (BS #1 and #2, BS #3 and #4) are parallel to the incident wave. Hence, there are only two virtual point sources in the implementation model. Even in the case of few virtual point sources, their arrangement can be set using the proposed method, as shown in Figure 12a,b.

In the linear array, illustrated in Figure 9b, the arrangement of the virtual point sources required to complete the BS correlation is determined when the incoming wave angle is 0°, as shown in Figure 12c. Then, *d*_14_ in the implementation model is set to 0.361 *λ*, which is 0.007 *λ* less than the desired distance, as shown in Figure 11b. The calculated spatial correlation *ρ*_14_ is 0.07, which is 0.02 larger than the desired BS correlation 0.05; however, the effect of this difference on the MIMO channel capacity is considered to be very small (see Figure 6). It is concluded from these analytical results that the initial phase matrix implemented on the MIMO-OTA apparatus representing the desired BS correlation can be generated using the proposed method, providing an OTA method for the evaluating the performance of the uplink channel from a MIMO mobile terminal with respect to the BS correlation characteristics.

## 5. Experimental Verification

### 5.1. Bidirectional Fading Emulator

**Figure 13:**
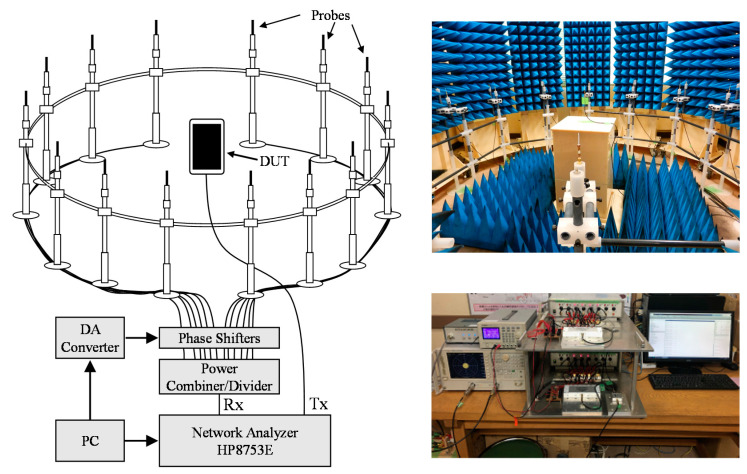
Two-dimensional bidirectional MIMO-OTA apparatus.

Figure 13 shows the configuration of the developed two-dimensional bidirectional fading emulator. To achieve a good multipath propagation environment, 14 scatterers are arranged at equal angular intervals on a circle of radius 1.2 m [34]. The probes comprise vertically polarized half-wavelength sleeve dipole antennas. The MIMO terminal is located at the center of the emulator. This fading emulator functions as an operating algorithm based on the same formulation described in [24]. The instruments, such as the network analyzer (Keysight, HP8753E), power combiner/divider (mini circuit, ZAPD-2-S+ and ZB8PD-252-S+), and phase shifter (mini circuit, JSPHS-23+), have bidirectional characteristics at the measurement frequency. This apparatus generates a fading environment by radio-frequency (RF) processing, and it can be implemented even if transmission and reception are reversed; thus, the performance of the uplink channel from the MIMO terminal can be evaluated.

To measure the uplink channel properties, a wave transmitted from a network analyzer is emitted from the MIMO terminal antenna and is then received at each probe. The received signals are controlled using phase shifters operated by a digital-to-analog converter to emulate the Rayleigh fading channel. These are compounded by a power combiner and then measured using a network analyzer. This apparatus has a high time correlation characteristic of approximately 0.995; thus, each channel response can be measured individually.

### 5.2. Measurement of the BS Correlation

In the first step of the investigation, the proposed method was evaluated via OTA testing with a uniform azimuthal angular power spectrum using a 4 × 4 correlation matrix according to the desired BS correlation using a two-dimensional bidirectional fading emulator. To evaluate the antenna performance on the terminal side, the MIMO terminal was placed at the center of the bidirectional fading emulator.

**Figure 14:**
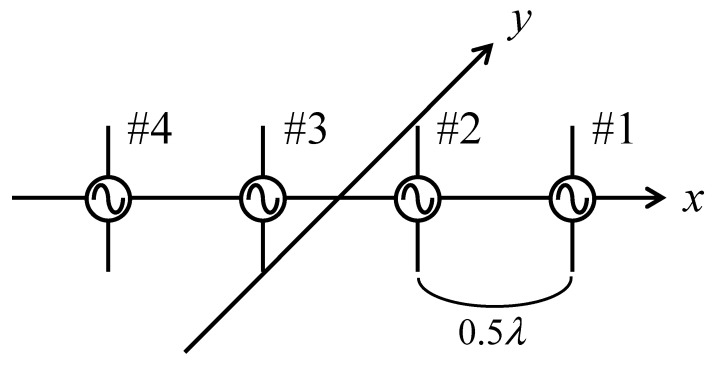
Configuration of the four-element MIMO terminal.

The arrangement of the MIMO terminal using a half-wavelength dipole antenna is a quasi-linear array with a half-wavelength spacing aligned along the *x*-axis, as presented in Figure 14. Table 2 lists the measurement and analytical conditions used to perform the OTA testing and Monte Carlo simulations. The frequency used in the measurement is 1.95 GHz, which is the center frequency of the uplink channel in the 2 GHz band in Japan. The polarization is assumed to be vertical because only vertically polarized dipole antennas are used in the bidirectional fading emulator.

**Figure 15:**
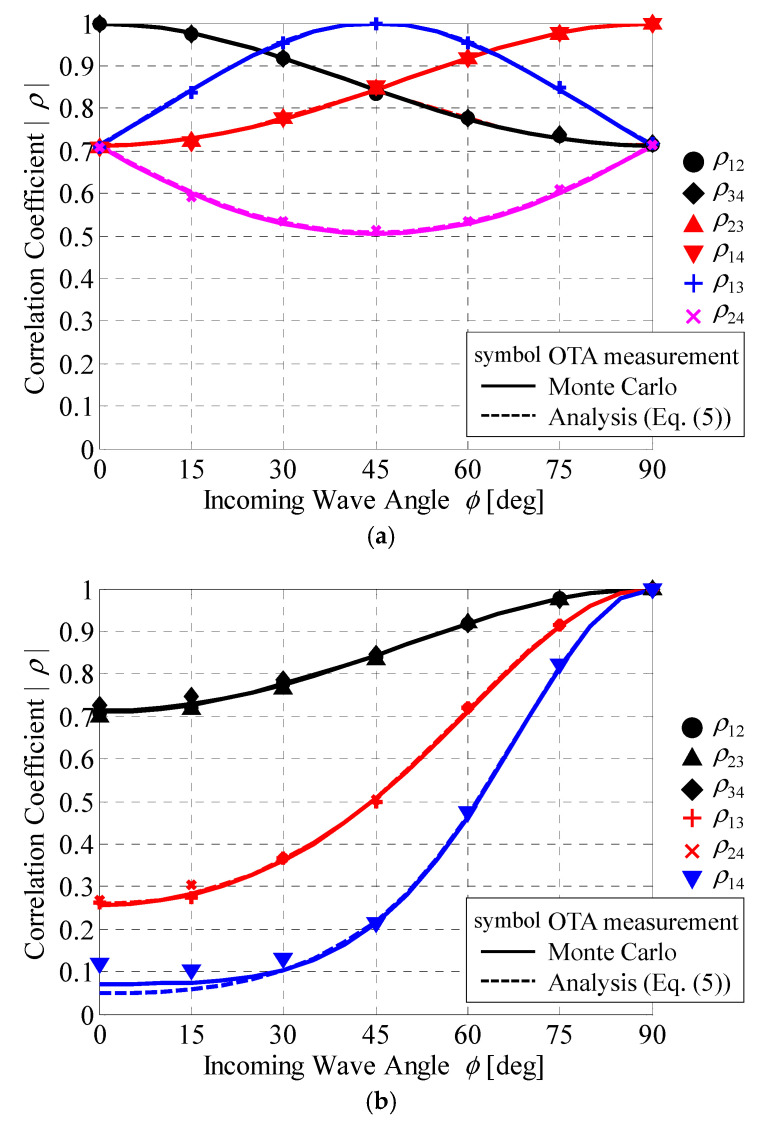
Characteristics of the BS correlation as a function of the incoming wave angle: (**a**) circular array; (**b**) linear array.

Figure 15 shows the BS correlation calculated using Equation (6) as a function of the incoming wave angle. The symbols are for OTA test results using the proposed method to realize the BS correlation, whereas the solid and broken curves indicate the analytical outcomes from the Monte Carlo simulations and the calculated correlation using Equation (5), respectively.

(6) $$\rho_{pq}=\frac{1}{4}\left(\frac{\left|h_{p1}h_{q1}{}^{*}\right|}{\sqrt{h_{p1}h_{p1}{}^{*}}\sqrt{h_{q1}h_{q1}{}^{*}}}+\frac{\left|h_{p2}h_{q2}{}^{*}\right|}{\sqrt{h_{p2}h_{p2}{}^{*}}\sqrt{h_{q2}h_{q2}{}^{*}}}+\frac{\left|h_{p3}h_{q3}{}^{*}\right|}{\sqrt{h_{p3}h_{p3}{}^{*}}\sqrt{h_{q3}h_{q3}{}^{*}}}+\frac{\left|h_{p4}h_{q4}{}^{*}\right|}{\sqrt{h_{p4}h_{p4}{}^{*}}\sqrt{h_{q4}h_{q4}{}^{*}}}\right)$$

where *h_pr_* indicates the channel response between the BS antenna #*p* and the MIMO terminal antenna #*r*, and the asterisk (*) denotes the complex conjugate.

It can be observed in Figure 15 that the experimental results obtained using the bidirectional fading emulator are in good agreement with both the Monte Carlo analysis results and the theoretical BS correlations obtained from Equation (5). As described in Section 4.3, the error in the BS correlation at *ϕ* = 0° in the linear array is owing to the error that occurs when creating the arrangement of the virtual point sources, as shown in Figure 12c. This confirms that the desired BS correlation in a 4 × 4 MIMO system can be achieved using the initial phase matrix, depending on the virtual point source arrangement.

### 5.3. Measurement of the 4 × 4 MIMO Uplink Channel Capacity

**Figure 16:**
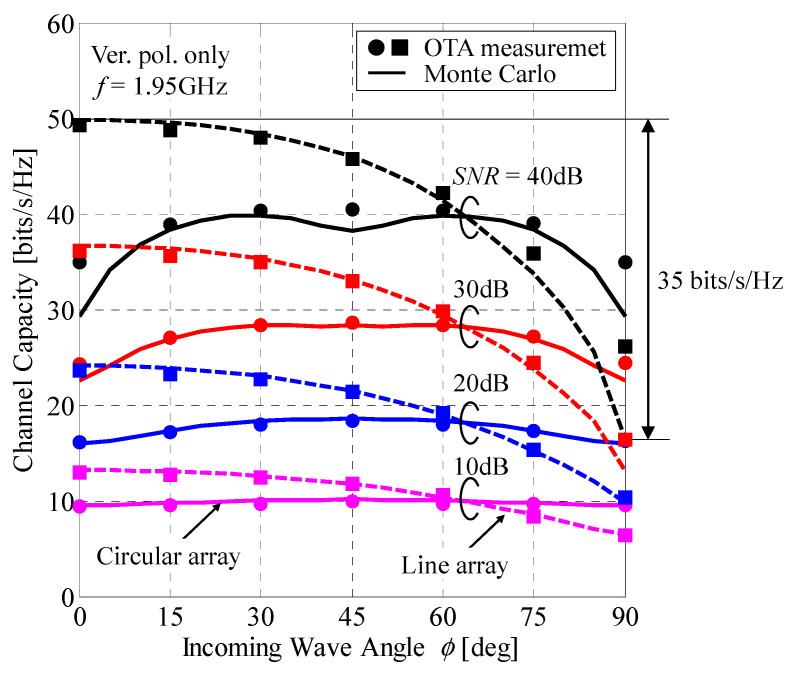
4 × 4 MIMO channel capacity as a function of the incoming wave angle.

The MIMO channel capacity was measured using a two-dimensional bidirectional fading emulator to evaluate the performance of the four-element MIMO terminal uplink channel. Figure 16 shows the 4 × 4 MIMO (four elements in the mobile terminal and four elements in the BS) channel capacity as a function of the incoming wave angle with the SNR as a parameter. The MIMO terminal illustrated in Figure 14 was placed at the center of the bidirectional fading emulator. The round and square symbols denote the OTA measurement results for the circular and linear arrays at the BS, respectively. The solid and broken curves indicate the Monte Carlo simulation results for the circular and linear arrays at the BS, respectively. The SNR was changed from 10 to 40 dB in 10 dB intervals [35]. The frequency was 1.95 GHz, and the XPR was set to 50 dB, which is equivalent to a vertically polarized propagation environment.

It can be observed from Figure 16 that the results measured via OTA testing are in good agreement with the analytical results obtained from the Monte Carlo simulations regardless of the BS arrangement and incoming wave angle. In the case of ϕ = 90° in the linear array, the measured channel capacity is slightly larger than the analytical value. A possible cause of this phenomenon is the BS correlation being insufficiently close to 1 because this apparatus cannot achieve a time correlation of 1. As shown in Figure 6, when the correlation coefficient approaches 1, the MIMO channel capacity deteriorates significantly. However, it is possible to evaluate the performance of the MIMO terminal because a 0.99 BS correlation can be achieved using this bidirectional fading emulator. Similarly, with a circular array at the BS, the same phenomenon was observed when the BS correlation was 1 depending on the incoming wave angle.

In the case of a circular array at the BS, shown in Figure 15, the six BS correlations do not have the same characteristic because the correlation increases or decreases depending on the incoming wave angle. Therefore, the MIMO channel capacity does not change significantly with respect to the incoming wave angle.

In the case of a liner array at the BS, the six BS correlations are inclined in the same direction depending on the incoming wave angle. Consequently, the MIMO channel capacity varies significantly with respect to the incoming wave angle compared with the case of a circular array. In particular, when the SNR is 40 dB, there is a difference of approximately 35 bits/s/Hz between the incoming wave angles of 0° and 90°.

Moreover, as shown in Figure 16, it can be seen that the channel capacity of the linear array is higher in the range of 0°–65° regardless of the SNR, whereas that of the circular array is higher in the range of 65°–90°. Comparing Figure 15a,b, the BS correlation for the linear array is lower than that of the circular array in the range of 0°–65°; thus, the channel capacity is higher. On the other hand, in the range of 65°–90°, the channel capacity is high because the BS correlation for the circular array is low. Considering the BS correlation at *ϕ* = 65°, there are three correlation values of approximately 0.93, two of approximately 0.76, and one of approximately 0.53 for both the circular and linear arrays. Therefore, the channel capacity is the same regardless of the arrangement of the BS antenna.

As described above, the fluctuations in the channel capacity characteristics with regard to the incoming wave angle, shown in Figure 16, can be considered via association with the BS correlation characteristics, shown in Figure 15. Thus, it is possible to use a bidirectional fading emulator to conduct an OTA evaluation of the uplink from a MIMO terminal antenna that considers the BS correlation characteristics.

## 6. Conclusions

This paper presents a method of implementing a 4 × 4 correlation matrix to realize the BS correlation characteristics in order to evaluate an uplink channel using bidirectional MIMO-OTA apparatus. The initial phases of the secondary sources were generated by arranging virtual point sources based on Jakes’ model according to the BS correlation. An examination of the effectiveness of the proposed method using bidirectional MIMO-OTA apparatus made it clear that the desired BS correlation characteristics can be achieved only by controlling the initial phase of the probe. Hence, the performance of the uplink from a MIMO terminal considering the BS correlation can be evaluated with high accuracy.

In this paper, only the vertical polarization component was examined. However, the MIMO terminal antenna is expected to have both vertical and horizontal polarization components. It is considered that the BS correlation can be controlled by setting the initial phases of the secondary sources for both the vertical and horizontal polarization components, which will be investigated in future work.

Furthermore, the practical propagation environment model for the uplink channel is assumed to be a three-dimensional channel model where radio waves propagate over the full-solid angle. In one of my previous studies [36], a methodology for controlling the BS correlation for a 2 × 2 MIMO system realized by a three-dimensional bidirectional fading emulator was reported. The results showed that the measured BS correlation of the uplink channel agreed well with the desired values, confirming the effectiveness of the proposed method for a three-dimensional channel model. The accuracy of the 4 × 4 correlation matrix will be improved because the flexibility of the virtual point source arrangement increases by placing the virtual point source in three-dimensional space. As a result, the difficulty of realizing all the BS correlations, as shown in Figure 12c, may be resolved.

The proposed OTA testing method will be applied to the evaluation of communication performance considering the correlation coefficient between the received signals for other wireless sensor networks, such as the Internet of Things (IoT) and emerging Vehicle-to-Everything (V2X) technologies [37,38,39]. To ensure the success of upcoming connected car systems, the author is currently developing a 256 × 256 MIMO antenna system that utilizes circular array beam steering technology [40]. However, in large-scale MIMO systems, there are some difficulties in terms of realizing OTA measurements. One of the difficulties is that the number of BS correlations is very large compared with the number of BS antennas. Another is that a large number of scatterers are necessary to create the full-rank channel matrix. Therefore, not only the scope of applications of the proposed method for uplink channels, but also OTA testing methods for downlink channels [41] need to be examined, which will be investigated in future work.

## Figures and Tables

**Table 1:** Novelty of the present work.

Method	Target	Issues Focused on
Reverberation chamber based method	Mobile terminal	Using a universal software radio peripheral [9] 2 × 2 MIMO, NLOS, 28 GHz [10]
Two-stage method	Mobile terminal	3D channel model, active antenna array [11] Non-Stationary channel model [12]
Multiple probe antenna based method	Mobile terminal	Cluster environment [13] Channel emulation technique [14] Plane-wave field synthesis [15] Bit error rate, IoT wireless device [16] 3D channel model, probe selection [17]
	Base station	Radiated testing of massive MIMO [18] Probe selection algorithm [19]

**Table 2:** Measurement and analytical conditions.

Frequency	1.95 GHz
Number of MIMO antennas	4
Arrangement of MIMO antenna	Quasi-liner array
MIMO antenna element	Half-wavelength dipole
Interval of MIMO antenna	0.5 *λ*
Number of BS antennas	4
Angular spread of the incident wave	1.5°
Number of probes	14
Traveling distance	5000
Number of samplings	5000
XPR	Vertical polarization only
Method of EM analysis	Method of moments

## Data Availability

Not applicable.

## Abbreviations

The following abbreviations are used in this manuscript:

MIMO	Multiple-input multiple-output
OTA	Over-the-air
BS	Base station
5G	Fifth-generation
3GPP	3rd Generation Partnership Project
CTIA	Cellular Telecommunication and Internet Association
XPR	Cross-polarization power ratio
i.i.d.	Independent and identically distributed
DUT	Device under test
NLOS	Non-line-of-sight
SNR	Signal-to-noise ratio
RF	Radio-frequency
IoT	Internet of Things
V2X	Vehicle-to-everything
**Notations**	
K	Number of secondary wave sources
$P_{mi}\left(t\right)$	Phase-shift of the carrier wave from the *i*-th scatterer according to the *m*-th BS antenna
$\phi_{i}$	Azimuth angle of the *i*-th scatterer
$\phi_{v}$	Direction of movement of the DUT antenna
$f_{D}$	Maximum Doppler frequency
v	Speed of the DUT antenna
λ	Wavelength of the carrier wave
$\varphi_{mi}$	Initial phase of the *i*-th scatterer with respect to the *m*-th BS antenna
ρ	Spatial correlation between the antennas
d	Distance between the isotropic antennas
$\Phi_{m}$	Initial phase matrix corresponding to BS #*m*
$\alpha_{mi}$	Geometrical phase difference between the virtual point source of BS #*m* and probe #*i*
|Δρ|	Difference between the calculated spatial correlation and the desired BS correlation
σ	Angular spread of the incident wave
ϕ	Relationships between conventional BS antenna arrangements and the incoming wave angle
$h_{pr}$	Channel response between the BS antenna #*p* and the MIMO terminal antenna #*r*
(*)	Complex conjugate
